
JOURNAL INFORMATION
==============================
Journal ID: Intereconomics

Intereconomics

EISSN: 1613-964X

ARTICLE INFORMATION
==============================
PMCID: PMC9755758
DOI: 10.1007/s10272-022-1083-0
Article ID: 1083
Article version: 1
Subjects: Editorial

Globalisation and Deglobalisation: The Impact and the Alternatives

Ghosh Jayati jghosh@umass.edu

grid.266683.f 0000 0001 2166 5835 College of Social and Behavioral Sciences, Depart. of Economics, University of Massachusetts Amherst, 315 Gordon Hall, Crotty Hall 412 North Pleasant Street, Amherst, MA 01002 USA
Electronic publication date: 2022 Dec 16
Print publication date: 2022
Volume: 57
Issue: 6
First page: 342
Last page: 343
Copyright: © The Author(s) 2022
License: Open Access: This article is distributed under the terms of the Creative Commons Attribution 4.0 International License (https://creativecommons.org/licenses/by/4.0/).
Open Access funding provided by ZBW — Leibniz Information Centre for Economics.
License URL: https://creativecommons.org/licenses/by/4.0/

Keywords: E20, F02, D30
issue-copyright-statement: © The Author(s) 2022

==============================

Global inequalities are now as extreme as they were at the peak of Western imperialism in the early 20th century, as the World Inequality Report 2022 makes clear (Chancel et al., 2022). The global income share of the poorest half of the world's population is only around half of what it was in 1820, before the great colonial divergence. Meanwhile, within-country inequalities have grown even faster, with income and wealth inequalities exploding at the very top of the distribution and private wealth almost wiping out publicly held assets in many countries. Things got worse during the COVID-19 pandemic, according to Oxfam (2022): the wealth of the top 1% increased dramatically and that of the ten richest men doubled, while 99% of humanity became poorer.

Extreme inequality of assets, incomes and opportunities does not only create unjust, unhealthy and unhappy societies – it actually kills people. In the past few years, millions of people have died after they contracted infectious diseases without getting vaccinated in time, because they did not get essential hospital care or oxygen when they needed it, because of shortages in underfunded public health systems, or because other illnesses and diseases were not treated in time.

Since then, and even more since the war in Ukraine and the subsequent increases in global prices of food and fuel, people have died of hunger without the means to buy food. Many have experienced despair and desperation at the loss of livelihood and this has been exacerbated by the fact that their governments could not or would not provide the social protection needed to survive the crisis.

Curiously, these terrible but avoidable outcomes were the result of both globalisation and deglobalisation. Decades of neoliberal globalisation involved a refashioning of global and national rules to favour large private capital, in the form of financial and real investments and greater "ease of doing business". It was associated with major privatisations: of public assets, of natural resources, of essential services (such as health, education and sanitation), of major amenities that were earlier seen as "natural monopolies" (like electricity) and even of knowledge (through intellectual property rights). It involved deregulation of other markets, leading to less protection of labour and the environment.

This led to sharp declines in public wealth across the world and extreme concentrations of assets in the hands of a few multinational corporations and rich individuals. Inequality also reduced mass consumption demand, making for lower levels of economic activity and therefore employment. Meanwhile, technological changes added to inequality by reducing demand for many types of workers.

The popular backlash against globalisation that is now being felt in most parts of the world reflects all this, as well as the geopolitical tensions that have created incentives for different trading and financial blocs to form. Unfortunately, the current deglobalisation also remains fundamentally neoliberal in its nature and inspiration, replacing a global covenant with a more petty nationalist one, but continuing to privilege big capital and the rich in favour of everyone else. This means it cannot be a real alternative for humanity, given our current truly existential challenges.

The basic issue is that today's most dire and intricate problems are global in nature: they do not respect passports and border controls. There is really no alternative to substantially increased international cooperation, especially on a few key issues.

To prevent catastrophic climate change, carbon dioxide and other greenhouse gas emissions have to be cut sharply – even though COP27 just showed us that most governments still refuse to recognise this and put short-term political interests above the most rational medium-term interests of their own people. Rich countries with high legacy emissions should obviously make the deepest cuts and transfer green technologies to the developing world without conditions, enabling the latter to also decarbonise rapidly. Funds for climate adaptation as well as for loss and damage are now essential. Any funds for this must be of sufficient quantity, and provided on global public investment lines rather than as "charity" from rich to poor countries (Expert Working Group on Global Public Investment, n.d.).

Broadening access to critical knowledge – whether to control this and possible future pandemics or to address climate change and other ecological challenges – is absolutely essential. This means a reworking of the Agreement on Trade-Related Aspects of Intellectual Property Rights to recognise this need, so that private corporate monopolies over essential knowledge and technologies cannot stop the world from progress or ensuring resilience. Building resilient and decentralised manufacturing capacity, including in the public sector, will be vital in order to deal effectively with future pandemics and other health crises, as well as "greening" our economic activities.

Similarly, global tax cooperation is a no-brainer. Simple rules that would make multinational companies pay the same rate of tax as purely domestic firms and ensure that the revenues are shared fairly between countries would reduce inequality and provide fiscally constrained developing economies with much-needed resources (Independent Commission for the Reform of International Corporate Taxation, 2020).

In the context of a looming external debt crisis that has already claimed some victims, an international sovereign debt resolution mechanism would reduce many developing countries' fiscal burdens (Ocampo, 2021), freeing up space for urgent spending. Regulating highly mobile cross-border finance, reining in credit rating agencies (Ghosh, 2021) and introducing conditions that make finance respond to social needs will also require international regulatory cooperation.

These are global strategies, not merely national. So a deglobalisation process that retains the worst and most unequal elements of the previous globalisation must be fought against. Instead, we must demand of our national governments a more progressive, just and equitable engagement with the world.

Jayati Ghosh, University of Massachusetts Amherst, USA.


References

Chancel, L., T. Piketty, E. Saez, G. Zucman et al. (2022), World Inequality Report 2022, World Inequality Lab.
Expert Working Group on Global Public Investment (n.d.), https://globalpublicinvestment.org/ (25 November 2022).
Ghosh, J. (2021, 16 March), Credit-Rating Agencies Could Derail Economic Recovery), Project Syndicate.
Independent Commission for the Reform of International Corporate Taxation (2020), The Global Pandemic, Sustainable Economic Recovery, and International Taxation, Report.
Ocampo, J. A. (2021, 10 August), The New-Old Sovereign-Debt Challenge, Project Syndicate.
Oxfam (2022, 17 January), Ten richest men double their fortunes in pandemic while incomes of 99 percent of humanity fall, Press Release.
